# Effect of probiotic administration during pregnancy on the functional diversity of the gut microbiota in healthy pregnant women

**DOI:** 10.1128/spectrum.00413-24

**Published:** 2024-04-30

**Authors:** Guangyu Ma, Hao Yan, Kian Deng Tye, Xiaomei Tang, Huijuan Luo, Zhe Li, Xiaomin Xiao

**Affiliations:** 1 Department of Obstetrics and Gynecology, The First Affiliated Hospital of Jinan University, Guangzhou, China; 2 Department of Obstetrics and Gynecology, The Fifth Affiliated Hospital of Guangzhou Medical University, Guangzhou, China; 3 Department of Obstetrics and Gynecology, The Third Affiliated Hospital of Sun Yat-Sen University, Guangzhou, China; Tainan Hospital, Ministry of Health and Welfare, Tainan, Taiwan

**Keywords:** functional prediction, gut microbiota, predictive metagenome profiling, pregnancy, probiotic

## Abstract

Our study aims to investigate the impact of probiotic consumption during pregnancy on gut microbiota functional diversity in healthy pregnant women. Thirty-two pregnant women were randomly assigned to two groups. The probiotic group (PG) consisted of pregnant women who consumed triple viable *Bifidobacterium longum*, *Lactobacillus delbrueckii bulgaricus*, and *Streptococcus thermophilus* tablets from the 32nd week of pregnancy until delivery. The functional profiles of the gut microbiota were predicted through high-throughput 16S rRNA sequencing results using PICRUSt software and referencing the Kyoto Encyclopedia of Genes and Genomes (KEGG) database. In the gut microbiota of the PG, the genera *Blautia* and *Ruminococcus*, as well as the species *Subdoligranulum*, showed significantly higher relative abundances compared to the control group (CG) (*P* < 0.05). At Level 1 of the KEGG signaling pathways, there was a significant reduction in the functional genes of the gut microbiota involved in Organismal Systems in the PG (*P* < 0.05). In Level 2 of the KEGG signaling pathways, there was a significant reduction in the functional genes of the gut microbiota involved in Infectious Disease in the PG (*P* < 0.05). In Level 3 of the KEGG signaling pathways, the PG exhibited a significant increase in the functional genes of the gut microbiota involved in ABC transporters, Oxidative phosphorylation, Folate biosynthesis, and Biotin metabolism (*P* < 0.05). The CG showed a significant increase in the functional genes related to Cysteine and methionine metabolism, Vitamin B6 metabolism, Tuberculosis, and *Vibrio cholerae* pathogenic cycle (*P* < 0.05). In conclusion, our findings suggest that probiotic supplementation during pregnancy has a significant impact on functional metabolism in healthy pregnant women.

**IMPORTANCE:**

Probiotics are considered beneficial to human health. There is limited understanding of how probiotic consumption during pregnancy affects the functional diversity of the gut microbiota. The aim of our study is to investigate the impact of probiotic consumption during pregnancy on the functional diversity of the gut microbiota. Our findings suggest that probiotic supplementation during pregnancy has a significant impact on functional metabolism. This could potentially open up new avenues for preventing various pregnancy-related complications. This also provides new insights into the effects of probiotic consumption during pregnancy on the gut microbiota and offers a convenient method for exploring the potential mechanisms underlying the impact of probiotics on the gut microbiota of pregnant women.

## INTRODUCTION

Probiotics are recognized by the World Health Organization as living microorganisms that can have a beneficial impact on human health (1). Probiotics primarily interact with the host by directly or indirectly influencing the composition of the gut microbiota (2). Interestingly, probiotics can be used to regulate imbalances in gut microbiota composition, reduce intestinal permeability, and lower the risk of diseases associated with immune or metabolic imbalances (3). Many clinical studies have begun to investigate the impact of probiotic supplementation during pregnancy on the gut microbiota. Research has shown that probiotics can help pregnant women control their weight during pregnancy and postpartum (4). There is evidence that probiotic supplementation during pregnancy can reduce the risk of preeclampsia, maintain insulin levels, and significantly decrease the risk of gestational diabetes (5–7). Probiotics can also control blood sugar levels and lipid metabolism in pregnant women with gestational diabetes (8). Supplementing probiotics during pregnancy can also reduce the occurrence of infant eczema and childhood allergic diseases (9). The use of probiotics during pregnancy, including species from the *Lactobacillus* and *Bifidobacterium* genera, is associated with minimal adverse effects on pregnancy outcomes and neonatal outcomes, and pregnant women tolerate them well (10–14).

Currently, research on gut microbiota diversity is quite mature, but the study of gut microbiota functional diversity is relatively limited in the field of microbiology. During pregnancy, the gut microbiota plays a pivotal role in maternal and fetal health (15–17). While the understanding of microbial composition has been expanding, it is equally crucial to focus on the functional diversity of the gut microbiota. Recent research suggests that the presence of specific microbial taxa and their functional potential contributes to maternal and infant health. The functional diversity of the gut microbiota is associated with several vital processes during pregnancy, including nutrient metabolism, immune system regulation, and the production of bioactive molecules (18–22). This encompassing the collective capacity of these microorganisms to carry out various metabolic and immunological functions holds significant importance during the unique physiological state of pregnancy. This interplay between the gut microbiota’s functional diversity and pregnancy outcomes underscores the need for a comprehensive exploration of these dynamics. Compared to gut microbiota composition, the functionality of the microbial community can better explain the impact of microorganisms on human health. Microbial community metabolic pathways are complex, and there are limited methods for their detection. Phylogenetic Investigation of Communities by Reconstruction of Unobserved States (PICRUSt) is a tool that predicts the functional diversity of bacterial communities based on high-throughput sequencing data (23, 24). PICRUSt has a relatively high accuracy in predicting the effects of drugs and diseases on microbial metabolism in animals and humans and has been used in the medical field for this purpose (25).

In our previous research, we found that probiotic supplementation during pregnancy had an impact on the gut microbiota and immune status of pregnant women (26). So, does probiotic supplementation during pregnancy affect the potential functional diversity of the gut microbiota in pregnant women? In this study, our objective is to investigate the hypothesis that probiotic consumption during pregnancy could potentially induce changes in the functional profiles of the gut microbiota in healthy pregnant women. So we conducted PICRUSt predictive analysis utilizing high-throughput 16S rRNA sequencing results and referenced the Kyoto Encyclopedia of Genes and Genomes (KEGG) database.

## MATERIALS AND METHODS

### Research object

The study project was authorized by the Institutional Review Board (IRB) for Human Subject Research at the First Affiliated Hospital of Jinan University (2019-011). Written informed consent was acquired from all the participants according to the HelsinkiDeclaration. Selection of pregnant women for regular prenatal care at the First Affiliated Hospital of Jinan University will follow specific criteria. Pregnant women will be required to sign an informed consent form before reaching 32 weeks of gestation. Inclusion criteria are as follows: (i) Chinese nationality, (ii) Singleton pregnancy, (iii) Pre-pregnancy body mass index between 18.5 and 24 kg/m², and (iv) Full-term pregnancy for their first pregnancy. Exclusion criteria are as follows: (i) Advanced maternal age; (ii) History of gastrointestinal disorders or a family history of gastrointestinal disorders; (iii) History of antibiotic use during pregnancy; (iv) Pre-existing medical conditions, including hypertension, diabetes, hyperthyroidism, rheumatic diseases, other autoimmune diseases, or endocrine disorders; (v) Pregnancy-related complications such as gestational hypertension or gestational diabetes; and (vi) History of blood transfusions, organ transplantation, or immunotherapy.

### Study design and sample collection

**Fig 1:**
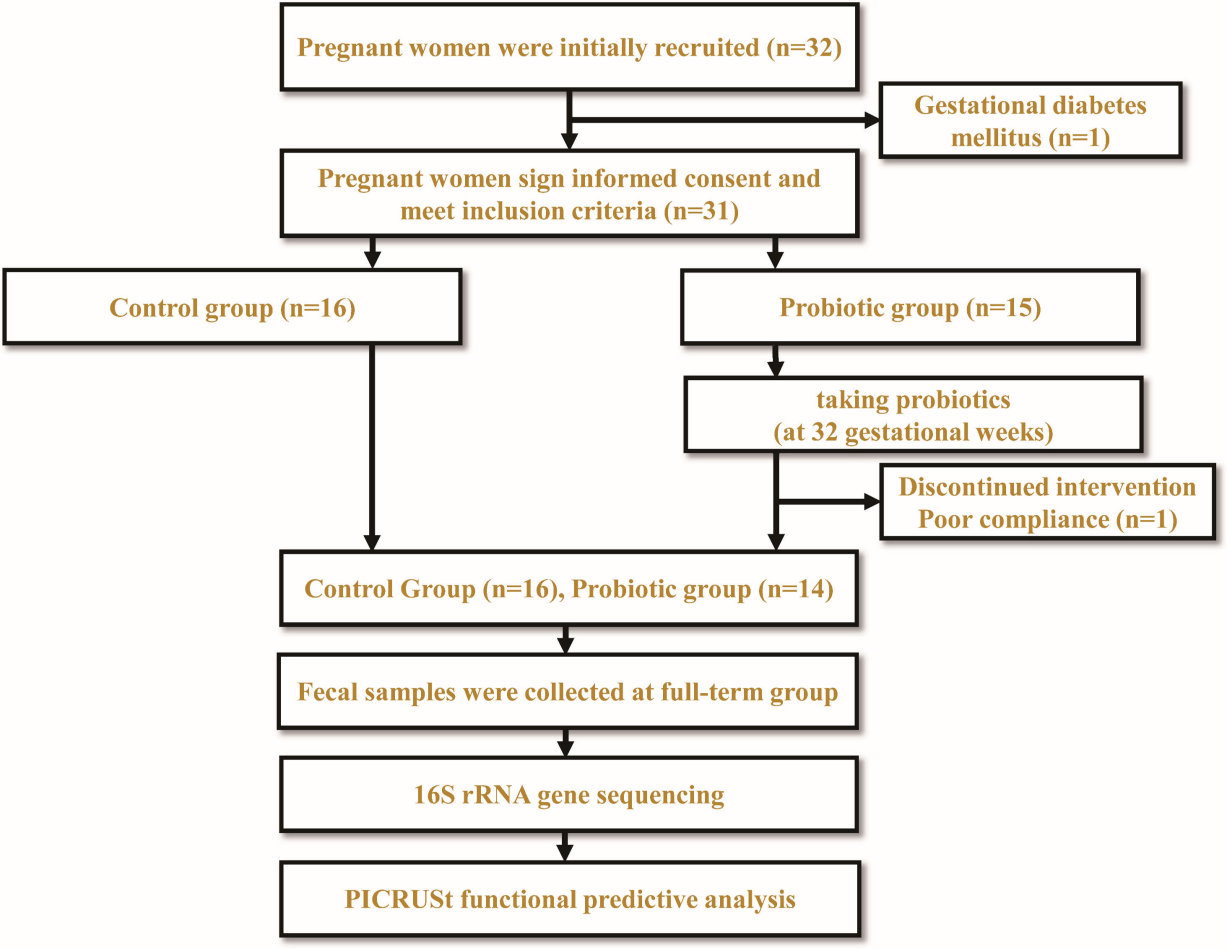
Experimental design workflow diagram.

Thirty-two pregnant women were initially recruited at The First Affiliated Hospital of Jinan University and were recruited before 32 weeks of gestation. They were randomly divided into two groups. However, one participant was later excluded due to the diagnosis of gestational diabetes mellitus during the third trimester, and another participant withdrew from the probiotic group (PG) before the study’s completion due to poor compliance. In the end, a total of 30 healthy pregnant women who meet the inclusion criteria will be enrolled in the study, with the control group (CG) consisting of 16 participants and the PG consisting of 14 participants. Pregnant women in the CG took no pills and if individuals were divided into PG, other probiotics were forbidden. In the PG, pregnant women will be instructed to take “triple viable *Bifidobacterium longum*, *Lactobacillus delbrueckii bulgaricus*, *and Streptococcus thermophilus tablets*” (0.5 g/tablet) twice daily. Each tablet contains no less than 0.5 × 10^7^ CFU of *B. longum*, no less than 0.5 × 10^6^ CFU of *Lactobacillus bulgaricus*, and no less than 0.5 × 10^6^ CFU of *S. thermophilus*. These supplements will be taken from the 32nd week of pregnancy until delivery. The CG, on the other hand, will not receive any probiotic supplementation. The experimental design is illustrated in Fig. 1. Fresh fecal samples from full-term pregnant women will be collected using sterile spoons. The samples will be obtained from the inner portion of the feces to prevent contamination with urine or other debris. They will be placed in disposable fecal specimen containers and promptly frozen at −80°C within thirty minutes (27, 28).

### Detection of fecal microbiota

The 16S rRNA V4 region sequencing was performed using the Ion S5 XL sequencing platform. The 16S V4 region primers used were 515F-806R. PCR amplification was carried out using the Phusion High-Fidelity PCR Master Mix with GC Buffer from New England Biolabs. Following PCR amplification, equal amounts of PCR products were combined and thoroughly mixed. The PCR products were then purified by 2% agarose gel electrophoresis using 1 × TAE buffer. After electrophoresis, the target bands were excised and recovered. Library construction was performed using the Ion Plus Fragment Library Kit 48 rxns from Thermo Fisher. Finally, sequencing was carried out using the Ion S5 XL sequencing platform, also from ThermoFisher.

### Data processing and analysis

To export FASTQ files from the Ion S5 XL sequencing platform’s raw data, the following data processing workflow is implemented as follows: Initially, the raw data are processed using the RS_ReadsOfinsert. 1 protocol within SMRT Portal version 2.3.0. This protocol is used to perform barcode splitting, separating the data based on the associated barcodes. Subsequently, chimera filtering is applied to eliminate chimeric sequences, resulting in a data set that is suitable for downstream analysis. Usearch is utilized for OTU clustering, with a clustering identity parameter set at 97%. All the filtered raw data are then realigned to OTU representative sequences, which are used to generate an OTU abundance table. The original OTU table is obtained through the development of custom scripts, and it is further categorized at different taxonomic levels. Finally, the HiSeq PE250 sequencing platform is employed to merge and filter reads, as well as perform OTU clustering.

### Statistical analysis

To prepare the data for further analysis, the PICRUSt platform is utilized to normalize the OTU abundance table of the samples. This normalization process corrects for variations in the copy numbers of the 16S marker gene within the genomes of different species. After normalization, the sequencing data from the samples are compared to the KEGG database. This comparison allows for the retrieval of metabolic pathway information from the KEGG database, which is then subjected to analysis. Linear discriminant analysis effect size (LEfSe) was performed, and the cladogram was depicted with the default parameter [Linear Discriminant Analysis (LDA) score = 4.0]. Functional categories' abundances are calculated based on the OTU abundance data, and correlations between differential microbial taxa and selected metabolic pathways are investigated. For the correlation analysis between differential microbial taxa and metabolic pathways, Spearman’s rank correlation test is applied. Statistical significance was set at *P* < 0.05.

## RESULTS

### Differential microbial analysis in the gut microbiota of the two groups of pregnant women using LEfSe

**Fig 2:**
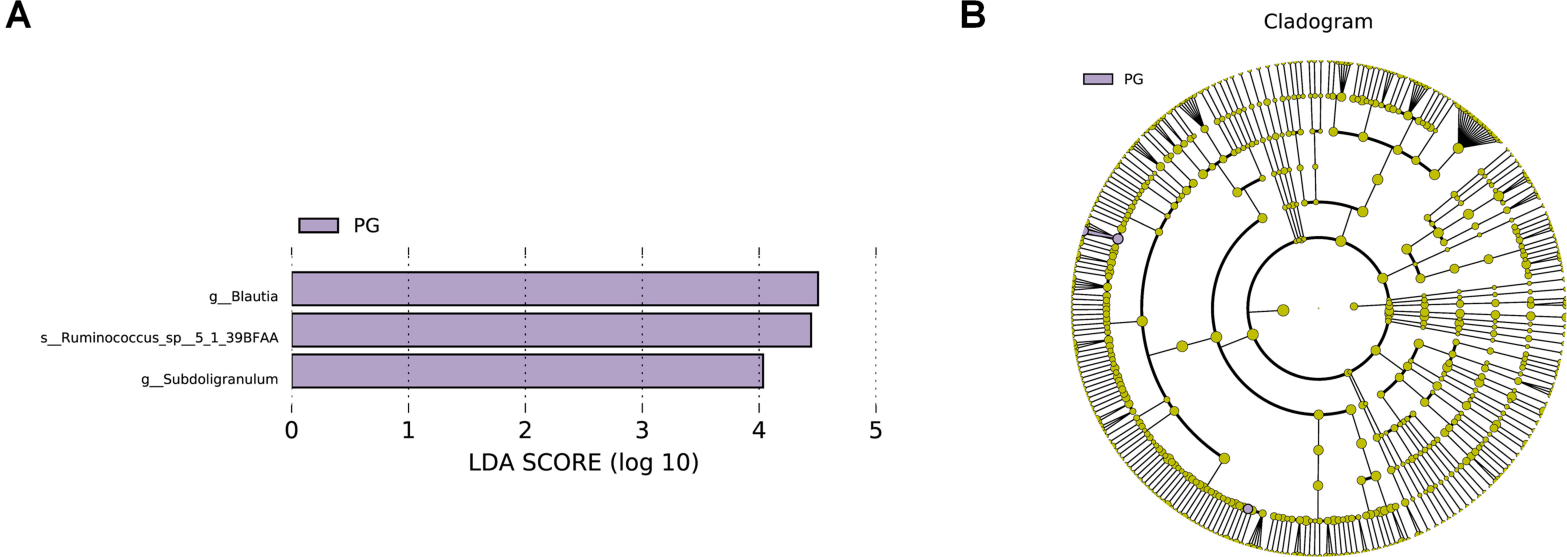
Distribution bar chart of LDA scores for differential microbes in the gut microbiota of two groups of pregnant women and the evolutionary branching diagram of differential microbes. (**A**) Distribution bar chart of LDA scores for differential microbes in the gut microbiota of two groups of pregnant women. Note: The bar chart in the LDA score distribution displays species with LDA scores greater than the set threshold, which are biomarkers indicating statistically significant differences between groups. The length of the bars represents the magnitude of the impact of differential species (i.e., LDA score). (**B**) Evolutionary branching diagram of differential microbes in the gut microbiota of two groups of pregnant women. Note: p__: Phylum, c__: Class, o__: Order, f__: Family, g__: Genus, s__: Species. CG: control group, PG: probiotic group.

**Fig 3:**
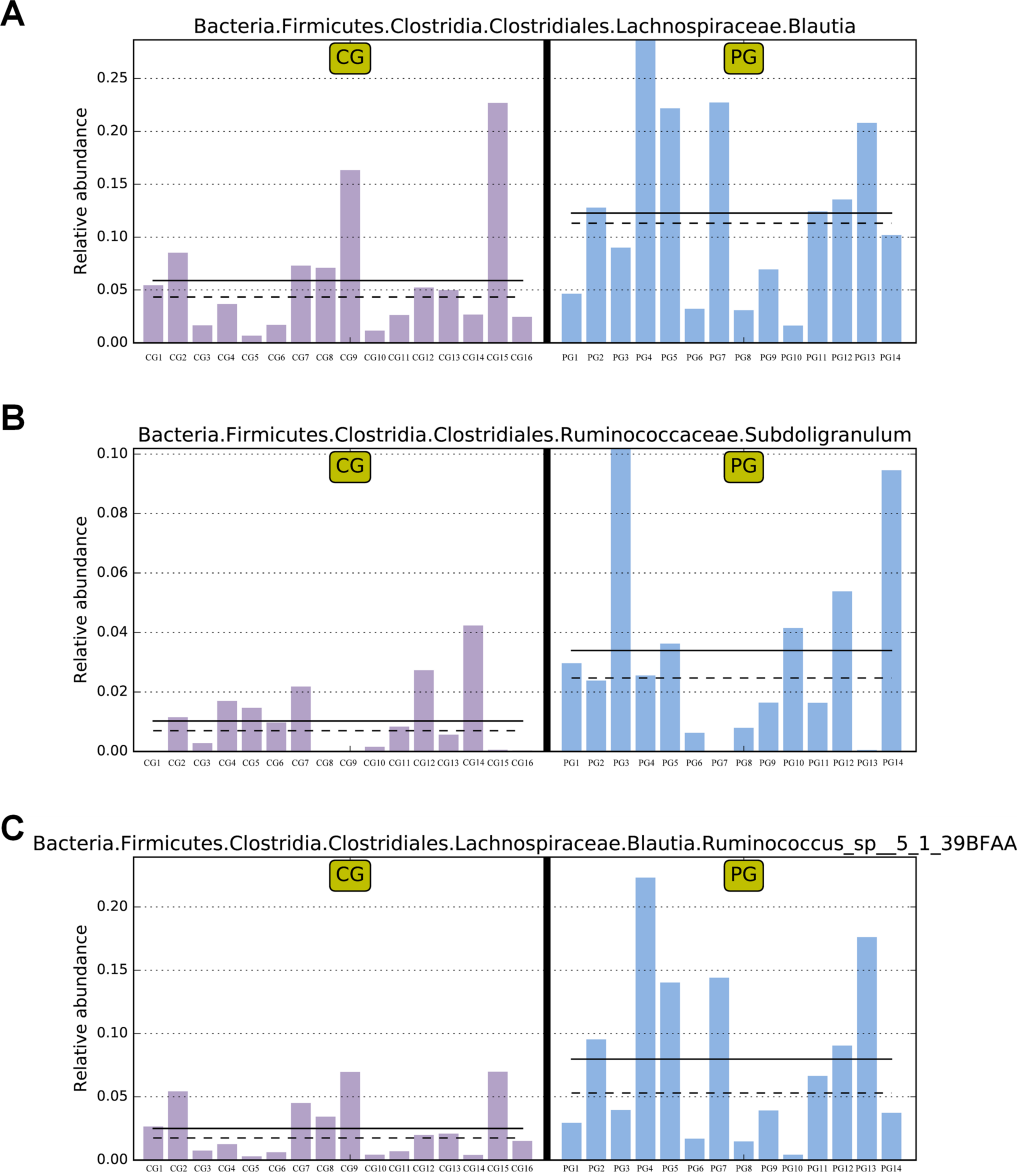
Comparative abundance of differential microbes in the gut microbiota of two groups of pregnant women. (**A**) Relative abundance comparison of genera *Blautia* in the gut microbiota of two groups of pregnant women. (**B**) Relative abundance comparison of genera *Subdoligranulum* in the gut microbiota of two groups of pregnant women. (**C**) Relative abundance comparison of species *Ruminococcus_sp__5_1_39BFAA* in the gut microbiota of two groups of pregnant women. Note: The abundance in the sample with the highest abundance is set as 1, and the abundance of the differential species in other samples is relative to the highest abundance sample. Solid lines and dashed lines represent the mean and median relative abundances of samples within the groups, respectively. If there is no bar in one of the groups, it indicates the absence of this differential species in that group. CG: control group, PG: probiotic group.

The LEfSe analysis allows for the comparison between two groups to identify species with significant differences in abundance between the groups. With an LDA threshold set at 4, the analysis filtered out differentially abundant microbial species between the two groups of pregnant women, as shown in Fig. 2. In the gut microbiota of pregnant women from the PG, the genera *Blautia* and *Subdoligranulum*, as well as the species *Ruminococcus_sp__5_1_39BFAA*, showed significantly higher relative abundances compared to the CG, and these differences were statistically significant (*P* < 0.05) (Fig. 2). The relative abundances of genera *Blautia*, and *Subdoligranulum*, as well as species *Ruminococcus_sp__5_1_39BFAA* in the gut microbiota of both groups of pregnant women are depicted in Fig. 3.

### PICRUSt functional prediction relative abundance bar chart

The analysis, based on the PICRUSt software, involves the prediction of functional metabolic pathways of gut microbiota from different samples. Sequencing data are compared to the KEGG database, and based on the database annotation results, functional information with relative abundances at various annotation levels is selected for each sample or group. This information is used to generate stacked bar charts depicting the relative abundance of functions at different annotation levels, allowing for a visual representation of the proportion of various functions in each sample.

**Fig 4:**
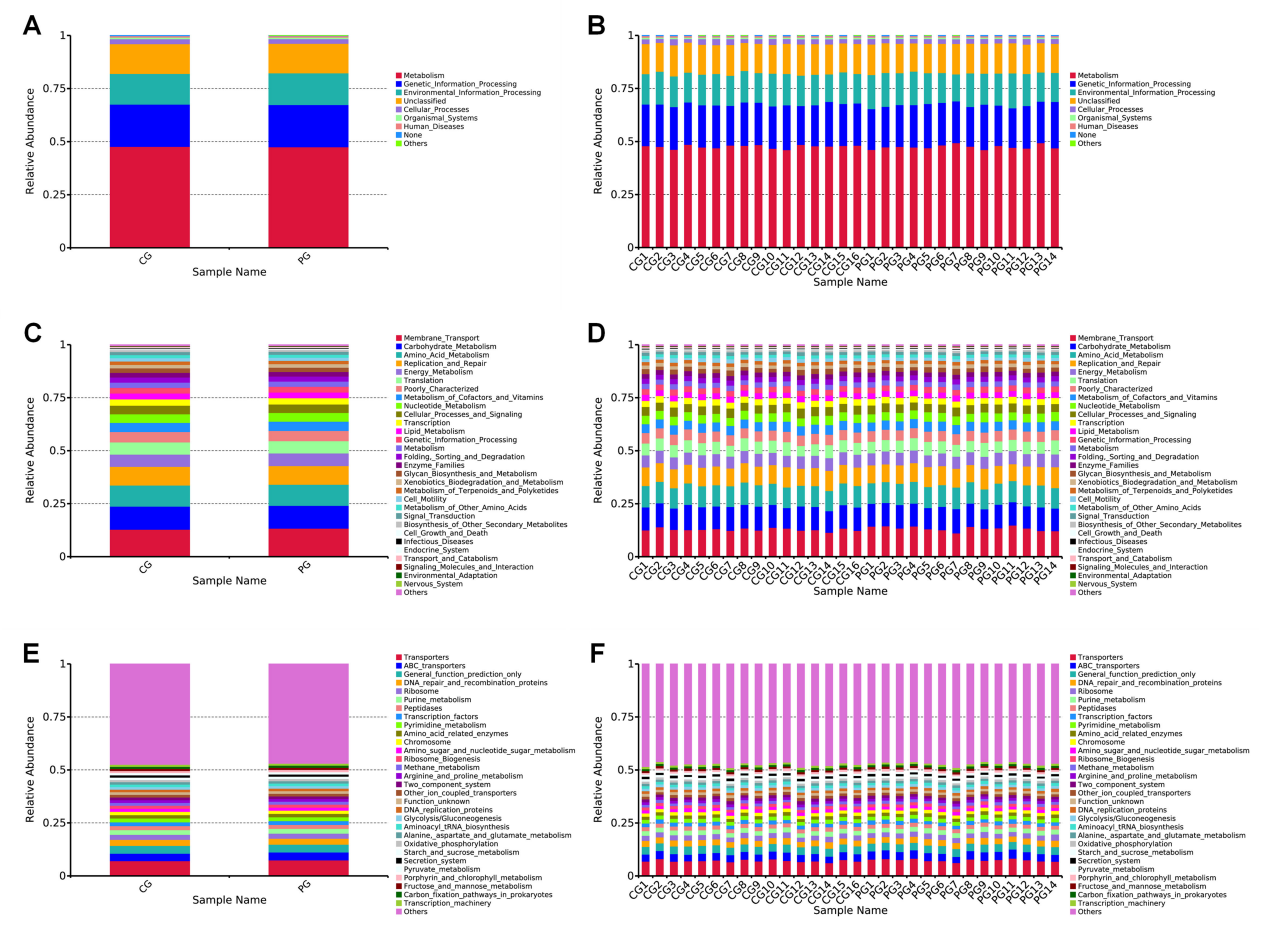
Stacked bar charts of PICRUSt functional metabolic relative abundance. (**A and B**) Level 1 PICRUSt functional metabolic relative abundance stacked bar charts. (**C and D**) Level 2 PICRUSt stacked bar charts showing the top 30 functional metabolic relative abundances. (**E and F**) Level 3 PICRUSt stacked bar charts showing the top 30 functional metabolic relative abundances. Note: The *x*-axis (Sample Name) represents sample names, and the *y*-axis (Relative Abundance) indicates the relative abundance. CG: control group, PG: probiotic group.

In terms of Level 1 metabolic pathways in the gut microbiota, both the CG and PG are involved in a total of six categories (Fig. 4A and B) (Tables S1 and S2). Among these, metabolism, genetic information processing, and environmental information processing are the major metabolic pathways for both CG and PG, ranking among the top 3. PG shows a decrease in Metabolism (48.70% in CG vs 47.53% in PG), Genetic information processing (19.89% in CG vs 19.86% in PG), Cellular processes (2.34% in CG vs 2.22% in PG), Organismal systems (0.72% in CG vs 0.68% in PG), and Human diseases (0.68% in CG vs 0.66% in PG) metabolic pathways compared to the CG group. However, the Environmental information processing (14.45% in CG vs 14.90% in PG) metabolic pathway is increased in the PG group compared to the CG group.

In terms of Level 2 metabolic pathways, both the CG and PG are involved in a total of 41 categories of biological metabolic pathways. We selected the top 30 functional information with the highest relative abundance for each sample or group at each annotation level, resulting in a stacked bar chart of functional relative abundance (Fig. 4C and D) (Tables S3 and S4). Among these pathways, Membrane Transport, Carbohydrate Metabolism, and Amino Acid Metabolism are the major metabolic pathways for both CG and PG, ranking among the top 3 for both groups. Compared to the CG, the use of probiotics leads to an increase in the following metabolic pathways in the PG: Membrane Transport (12.86% in CG vs 13.38% in PG), Amino Acid Metabolism (9.919% in CG vs 9.922% in PG), Energy Metabolism (5.80% in CG vs 5.94% in PG), Translation (5.74% in CG vs 5.78% in PG), Metabolism of Cofactors and Vitamins (4.32% in CG vs 4.38% in PG), Nucleotide Metabolism (4.07% in CG vs 4.09% in PG), Genetic Information Processing (2.71% in CG vs 2.72% in PG), Metabolism (2.408% in CG vs 2.411% in PG), Xenobiotics Biodegradation and Metabolism (1.62% in CG vs 1.66% in PG), and Cell Growth and Death (0.50% in CG vs 0.51% in PG).

In Level 3 metabolic pathways, we selected the top 30 functional information with the highest relative abundance for each sample or group at each annotation level, resulting in a stacked bar chart of functional relative abundance (Fig. 4E and F) (Tables S5 and S6). Among these pathways, Transporters, ABC transporters, and A General function prediction only are the major metabolic pathways for both CG and PG, ranking among the top three for both groups. Compared to the CG, the use of probiotics leads to an increase in the following metabolic pathways in the PG: Transporters (7.15% in CG vs 7.49% in PG), ABC transporters (3.51% in CG vs 3.76% in PG), General function prediction only (3.65% in CG vs 3.62% in PG), Ribosome (2.38% in CG vs 2.40% in PG), Purine metabolism (2.23% in CG vs 2.26% in PG), Transcription factors (1.78% in CG vs 1.79% in PG), Amino acid related enzymes (1.53% in CG vs 1.56% in PG), Ribosome Biogenesis (1.432% in CG vs 1.435% in PG), Methane metabolism (1.29% in CG vs 1.36% in PG), Aminoacyl tRNA biosynthesis (1.20% in CG vs 1.21% in PG), Oxidative phosphorylation (1.05% in CG vs 1.10% in PG), Porphyrin and chlorophyll metabolism (0.97% in CG vs 1.07% in PG), Fructose and mannose metabolism (1.00% in CG vs 1.01% in PG), and Carbon fixation pathways in prokaryotes (0.98% in CG vs 0.99% in PG).

### PICRUSt function prediction Venn diagram and PCA analysis

**Fig 5:**
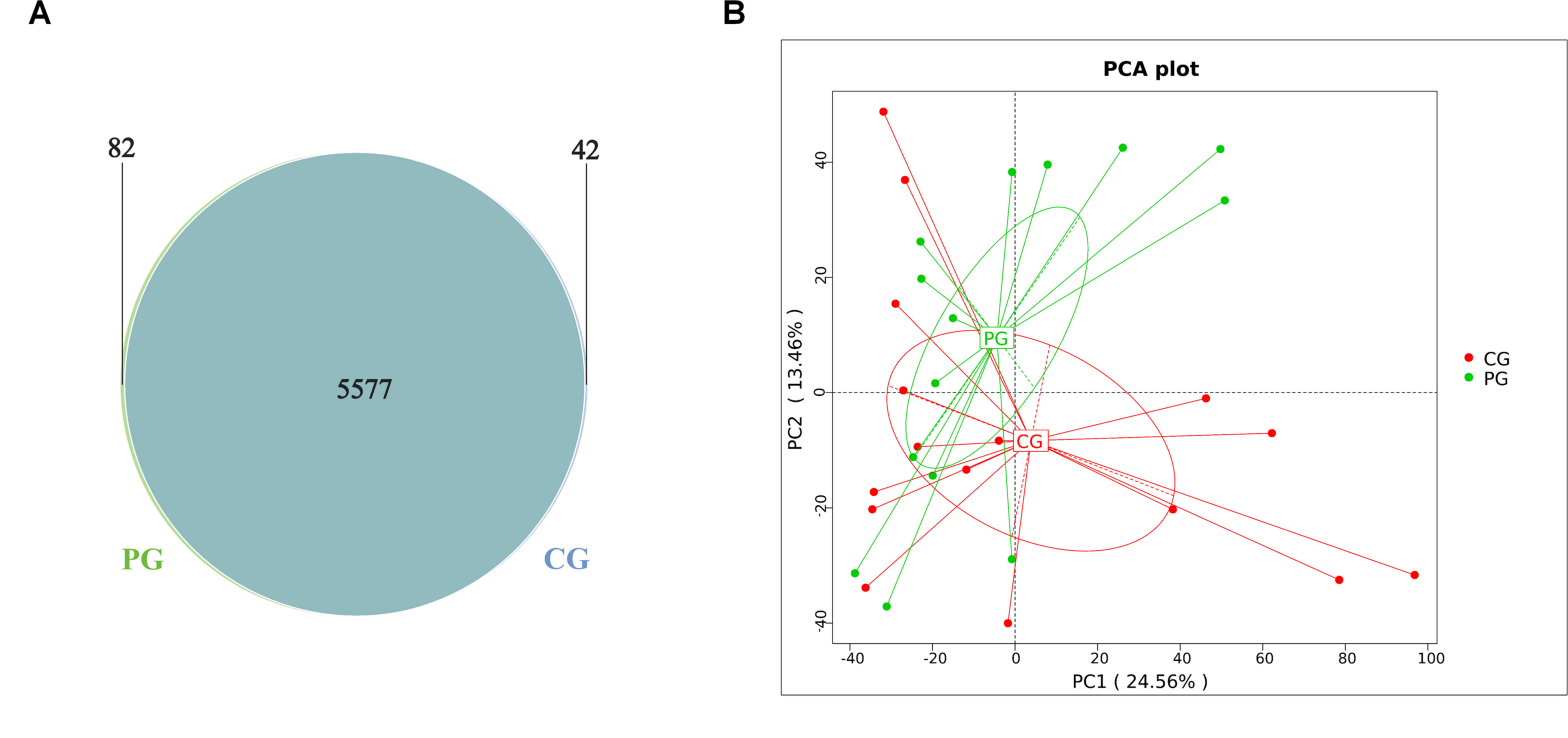
PICRUSt functional prediction Venn diagram and PCA analysis. (**A**) Venn diagram depicting the distribution of functional genes in the gut microbiota. (**B**) PCA analysis based on the composition of functional genes in the gut microbiota. CG: control group, PG: probiotic group.

To observe the distribution of gene numbers between the CG and PG and analyze the shared and unique gene information, a Venn diagram was constructed. It revealed that the CG and PG shared 5577 genes, while the CG had 42 unique genes, and the PG had 82 unique genes (Fig. 5A). Principal component analysis (PCA) was performed based on the abundance statistics of functional annotations from the database. In PCA, samples with similar functional compositions are closer to each other in the plot. The first principal component contributed to 24.56% of the sample differences, while the second principal component contributed to 13.46%. Compared to the CG, probiotic treatment resulted in significant differences in functional genes in the gut microbiota of both groups (Fig. 5B).

### PICRUSt analysis of intergroup functional metabolic differences in gut microbiota

**Fig 6:**
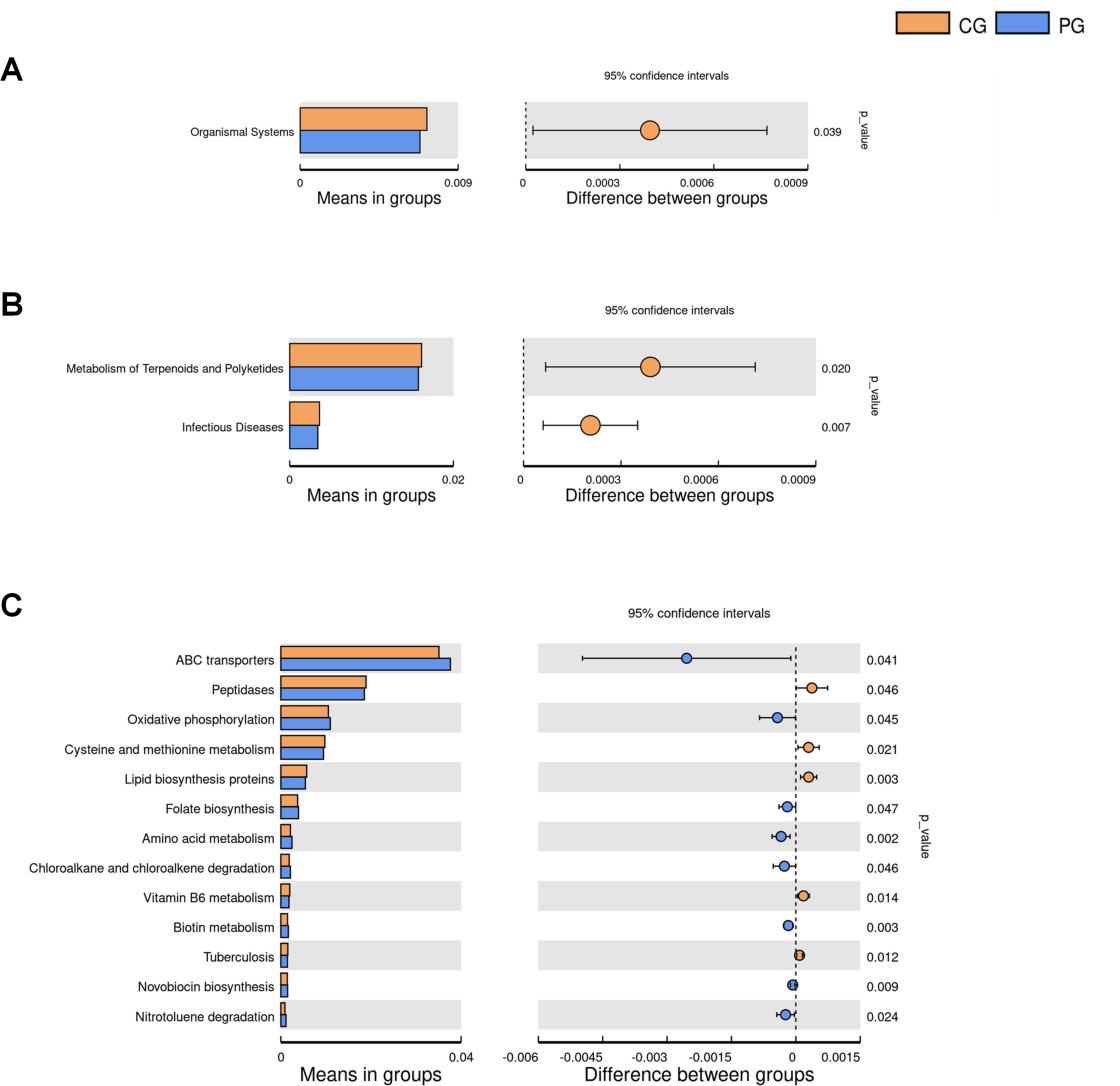
Bar charts of gut microbiota inter-group functional metabolic differences analyzed using PICRUSt. (**A**) Bar chart depicting gut microbiota functional metabolic differences at the KEGG Level 1 signal pathway. (**B**) Bar chart illustrating gut microbiota functional metabolic differences at the KEGG Level 2 signal pathway. (**C**) Bar chart showing gut microbiota functional metabolic differences at the KEGG Level 3 signal pathway. Note: Different colors in the figure represent different groups. On the left side are the KEGG categories with significant differences between groups and their proportions in each group. On the right side are the confidence intervals and *P* values for inter-group differences. The leftmost endpoint of each circle represents the lower limit of the 95% confidence interval for the mean difference, the rightmost endpoint represents the upper limit of the 95% confidence interval for the mean difference, and the center of the circle represents the mean difference. CG: control group, PG: probiotic group.

In the KEGG Level 1 signal pathways, which are divided into six categories including Metabolism, Genetic information processing, Environmental information processing, Cellular processes, Organismal systems, and Human diseases, significant differences in gut microbiota functional genes were determined by filtering KEGG Level 1 pathways with *P* < 0.05 (Fig. 6A) (Table 1). Probiotic intervention resulted in a significant decrease in gut microbiota functional genes related to Organismal systems in the PG, with statistical significance (*P* < 0.05). At the KEGG Level 2 signal pathways, two significant differences in gut microbiota functional genes were identified by filtering KEGG Level 2 pathways with *P* < 0.05 (Fig. 6B) (Table 2). Compared to the CG, probiotic treatment in the PG group led to a significant decrease in gut microbiota functional genes related to Metabolism of Terpenoids and Polyketides and Infectious Diseases (*P* < 0.05). In the KEGG Level 3 signal pathways, 18 significant differences in gut microbiota functional genes were determined by filtering KEGG Level 3 pathways with *P* < 0.05 (Fig. 6C) (Table 3). Compared to the CG, probiotic treatment during pregnancy in the PG resulted in a significant increase in gut microbiota functional genes related to ABC transporters, Oxidative phosphorylation, Folate biosynthesis, Amino acid metabolism, Chloroalkane and chloroalkene degradation, Biotin metabolism, Novobiocin biosynthesis, Nitrotoluene degradation, N-Glycan biosynthesis, Chlorocyclohexane and chlorobenzene degradation, and Ether lipid metabolism, with statistical significance (*P* < 0.05). In comparison to the PG group, the CG showed a significant increase in gut microbiota functional genes related to Peptidases, Cysteine and methionine metabolism, Lipid biosynthesis proteins, Vitamin B6 metabolism, Tuberculosis, *Vibrio cholerae* pathogenic cycle, and Flavone and flavonol biosynthesis, with statistical significance (*P* < 0.05).

**TABLE 1:** Comparison table of microbial metabolic functional differences at KEGG Level 1

KO functional categories	CG		PG		*P* Value
	Mean	SD	Mean	SD	
Organismal systems	0.007222	0.000363	0.006826	0.000580	0.038572

**TABLE 2:** Comparison table of microbial metabolic functional differences at KEGG Level 2

KO functional categories	CG		PG		*P* Value
	Mean	SD	Mean	SD	
Metabolism of terpenoids and polyketides	0.016115	0.000403	0.015725	0.000451	0.019507
Infectious diseases	0.003643	0.000233	0.003437	0.000150	0.007347

**TABLE 3:** Comparison table of microbial metabolic functional differences at KEGG Level 3

KO functional categories	CG		PG		*P* value
	Mean	SD	Mean	SD	
ABC transporters	0.035086	0.002583	0.037631	0.003667	0.040747
Peptidases	0.018914	0.000608	0.018540	0.000345	0.045741
Oxidative phosphorylation	0.010548	0.000498	0.010976	0.000598	0.044560
Cysteine and methionine metabolism	0.009765	0.000238	0.009468	0.000386	0.021199
Lipid biosynthesis proteins	0.005737	0.000178	0.005439	0.000295	0.003470
Folate biosynthesis	0.003727	0.000248	0.003925	0.000270	0.047119
Amino acid metabolism	0.002130	0.000224	0.002474	0.000316	0.002435
Chloroalkane and chloroalkene degradation	0.001852	0.000366	0.002118	0.000332	0.045856
Vitamin B6 metabolism	0.001983	0.000130	0.001806	0.000216	0.014341
Biotin metabolism	0.001485	9.48E−05	0.001660	0.000167	0.002501
Tuberculosis	0.001548	9.15E−05	0.001462	8.53E−05	0.012121
Novobiocin biosynthesis	0.001438	5.90E−05	0.001511	7.89E−05	0.009350
Nitrotoluene degradation	0.000911	0.000177	0.001151	0.000328	0.024181
*Vibrio cholerae* pathogenic cycle	0.000669	7.23E−05	0.000605	6.93E−05	0.019231
N-Glycan biosynthesis	0.000194	4.08E−05	0.000233	5.67E−05	0.041231
Chlorocyclohexane and chlorobenzene degradation	0.000119	4.35E−05	0.000173	7.06E−05	0.022842
Ether lipid metabolism	4.47E−05	2.56E−05	8.80E−05	4.39E−05	0.004102
Flavone and flavonol biosynthesis	6.69E−05	3.00E−05	4.59E−05	2.31E−05	0.039905

### Correlation analysis between differential microbial communities and differential metabolic pathways between the two groups

**Fig 7:**
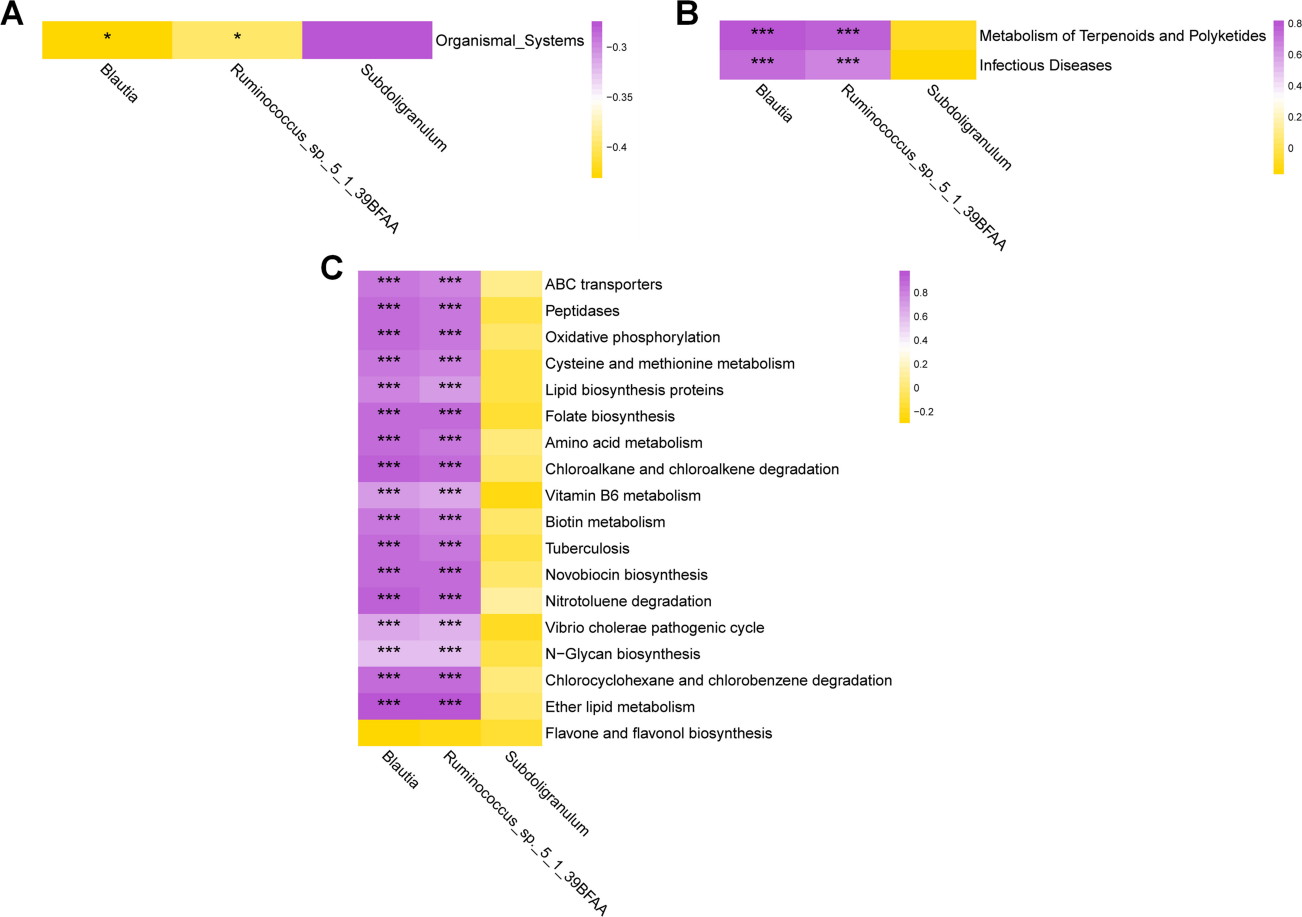
Heatmap of correlation between gut microbiota and metabolic functions. The labels at the bottom represent the names of gut microbial communities, and the labels on the right represent the names of metabolic functions. Purple indicates a positive correlation, while yellow indicates a negative correlation. *P < 0.05, ***P < 0.001.

In the correlation analysis between differential microbial communities and differential metabolic pathways between the two groups, at the KEGG Level 1 signal pathway, *Blautia* and *Ruminococcus_sp__5_1_39BFAA* exhibited a negative correlation with the Organismal systems metabolic pathway (Fig. 7A). At the KEGG Level 2 signal pathway, *Blautia* and *Ruminococcus_sp__5_1_39BFAA* showed a positive correlation with the Infectious diseases metabolic pathway, and they also exhibited a positive correlation with the Metabolism of Terpenoids and Polyketides metabolic pathway (Fig. 7B). At the KEGG Level 3 signal pathway, *Blautia* exhibited a positive correlation with metabolic pathways such as ABC transporters, while *Ruminococcus_sp__5_1_39BFAA* displayed a positive correlation with metabolic pathways including ABC transporters (Fig. 7C).

## DISCUSSION

Pregnancy induces various physiological changes in women, including alterations in the gut microbiota (29, 30). The maternal gut microbiota plays a crucial role in fetal development and programming (31). Additionally, the composition and diversity of the maternal gut microbiota can significantly influence the establishment of the infant gut microbiota and have long-term health outcomes for the offspring (32–34). Although probiotics have been shown to cause variation in the composition of the gut microbiota (35), their effects on pregnant women and their functional diversity remain unclear. Previous work showed that probiotic could work in about 2–4 weeks (36–38). To investigate the hypothesis that probiotic consumption during pregnancy could potentially induce changes in the functional profiles of the gut microbiota in healthy pregnant women, we executed this study.

During pregnancy, probiotic supplementation has various benefits, including the prevention of conditions such as preeclampsia, gestational diabetes, and the prevention of vaginal infections, as well as the regulation of maternal and infant weight (39). Studies emphasize the critical role of the gut microbiota in synthesizing vitamins B and K and metabolizing bile acids (40). Some researchers transplanted the gut microbiota from early and late pregnancy pregnant women into germ-free mice. They found that mice with the gut microbiota from late pregnancy women showed higher weight and had more possibility of developing insulin resistance than those with early maternal gut microbiota (41). Some suggest that the late pregnancy gut microbiota composition is similar to that of individuals with obesity (29). These findings underscore the significant role of the gut microbiota in metabolic aspects during pregnancy.

Nowadays, few studies focus on how probiotics affect the functional diversity of the gut microbiota in pregnant women during pregnancy. In this study, in order to investigate whether probiotic supplementation during pregnancy influences the functional metabolism of the gut microbiota in pregnant women, we applied the PICRUSt to predict functions based on 16S sequencing data and the KEGG database. The results indicated that there were differences in metabolic functions of the gut microbiota between pregnant women in the CG and PG. Probiotic supplementation during pregnancy can alter various metabolic pathways in the KEGG signaling pathways, mainly involving metabolism, genetic information processing, environmental information processing, cellular processes, organismal systems, and human diseases. At the KEGG Level 2 pathways, probiotics led to a significant decrease in microbial gene functions related to infectious diseases in the PG. This suggests that probiotic supplementation during pregnancy may reduce the risk of infectious diseases in pregnant women and play an important role in resisting pathogen invasion. At the KEGG Level 3 signaling pathways, probiotic supplementation led to a significant increase in various microbial gene functions in the gut of pregnant women. For example, ABC transporters, Oxidative phosphorylation, Folate biosynthesis, and Biotin metabolism were significantly increased. ABC transporters are membrane proteins that facilitate the transport of various substrates, promoting the uptake and release of various substrates, allowing them to participate in various cellular processes such as nutrient absorption, secretion of cell waste, maintenance of osmotic pressure, lipid transport, and transport of biomacromolecules (42). Studies on the predicted gut microbiota functions of pregnant women with gestational diabetes have shown that the microbial gene function related to ABC transporters in gestational diabetes is significantly lower than in normal pregnant women (43). Although the potential mechanisms of ABC transporters in gestational diabetes are not clear, studies have found a decrease in ABC transporters in diabetic mice (44). This study found that probiotics significantly increased ABC transporters, which may suggest that probiotic supplementation during pregnancy could prevent the occurrence of gestational diabetes, providing a new mechanism for the prevention of diabetes during pregnancy with probiotic supplementation. Oxidative phosphorylation is involved in maintaining mitochondrial function. The results of this study suggest that probiotic supplementation may promote oxidative phosphorylation. Mitochondria regulate cell metabolism, and studies have found a decrease in oxidative phosphorylation and mitochondrial respiration in the placentas of preeclampsia pregnancies (45). Probiotic supplementation during pregnancy may potentially prevent preeclampsia. Biotin is a water-soluble vitamin that plays a crucial role in the metabolism of fatty acids, amino acids, glucose, and also exerts various biological effects through histone modification, such as immune function and fetal growth (46). Studies have shown that maternal biotin deficiency during pregnancy may increase the risk of preterm birth or fetal growth restriction (47). Animal experiments have shown a direct relationship between maternal biotin deficiency during pregnancy and fetal growth retardation, congenital fetal abnormalities, and embryo death (48–51). In pregnant women with severe morning sickness, biotin deficiency was found, and the severity of morning sickness was negatively correlated with serum biotin levels (52). This study suggests that probiotics can increase Biotin metabolism, which implies that the use of probiotics during pregnancy may play an important role in preventing embryonic growth retardation and fetal malformations. Research indicates that Folate biosynthesis provides substrates for the biosynthesis of DNA, RNA, proteins, and lipids, which are essential for processes such as cell replication and differentiation (53). Folate plays a critical role during pregnancy, as an adequate level of folate during pregnancy can increase the chances of successful fertilization, improve implantation rates, reduce the risk of neural tube defects, and increase the rate of live births (54–56). During pregnancy, the demand for folate increases due to fetal growth and increased red blood cell production (57). Folate deficiency is a severe risk factor for neural tube defects and other congenital developmental abnormalities in the fetus (58). The results of this study show that probiotics can promote the intestinal microbial gene function of Folate biosynthesis. Probiotics play an important role in promoting the synthesis of folate in the body and may have beneficial effects on pregnant women and infants. In addition, it has been observed that probiotics supplementary affected other metabolic functions of the intestinal microbiota, including amino acid metabolism, chloroalkane and chloroalkene degradation, novobiocin biosynthesis, nitrotoluene degradation, N-Glycan biosynthesis, chlorocyclohexane and chlorobenzene degradation, and ether lipid metabolism. Specifically, the increase in amino acid metabolism may indicate heightened activity in amino acid metabolism in pregnant women, which related to their nutritional intake and metabolic status, as well as to probiotic intake. The heightened chloroalkane and chloroalkene degradation, chlorocyclohexane and chlorobenzene degradation may reflect an enhanced metabolic capacity of the gut microbiota toward environmental chlorinated compounds. The increased novobiocin biosynthesis and nitrotoluene degradation suggested that certain bacteria in the gut are initiating novobiocin synthesis and degrading nitrotoluenes. The increase in N-Glycan biosynthesis may be related to the demand for glycoprotein synthesis within pregnant women. The heightened ether lipid metabolism may reflect microbial involvement in ether lipid metabolism processes following probiotic intake. Overall, these results indicated that probiotic intake may lead to adjustments in the metabolic functions of the gut microbiota, but further research is needed to confirm the specific mechanisms and impacts.

We also found that the use of probiotics at KEGG Level 3 signaling pathways led to a significant reduction in various microbial gene functions in the intestines of pregnant women. These reductions included pathways such as Cysteine and methionine metabolism, Vitamin B6 metabolism, Tuberculosis, and *Vibrio cholerae* pathogenic cycle. Elevated plasma concentrations of cysteine have been associated with cardiovascular diseases (59, 60). Cysteine is also related to endothelial dysfunction (61). It has been reported that elevated maternal cysteine or homocysteine concentrations in preeclampsia may have adverse effects on placental function or the fetus (61–63). This study found that probiotic supplementation can reduce Cysteine metabolism, suggesting that probiotics may play an important role in preventing cardiovascular diseases and preeclampsia during pregnancy. Vitamin B6 is a water-soluble vitamin that plays a crucial role in many metabolic processes in the human body and contributes to the development and function of the nervous system. Research has shown that supplementing with vitamin B6 during pregnancy can reduce the severity of nausea during pregnancy (64, 65). Vitamin B6 also plays a role in preventing preeclampsia and premature birth, and routine supplementation of vitamin B6 during pregnancy is often recommended to reduce the risk of preeclampsia and premature birth (66, 67). Furthermore, maternal supplementation with vitamin B6 during pregnancy can reduce the risk of cardiovascular abnormalities in offspring (68). However, this study found an increase in the intestinal microbial functional genes related to Vitamin B6 metabolism in the CG. The reasons for the increased Vitamin B6 metabolism require further investigation in future studies. Additionally, it was observed that disease-related pathways, such as Tuberculosis and *Vibrio cholerae* pathogenic cycle, were reduced in the PG compared to the CG. This result suggested that within the microbial gene functions observed in the CG, pathways related to tuberculosis and *Vibrio cholerae* pathogenic cycle were relatively more active or expressed compared to the PG. Probiotic supplementary showed negative correlation with disease-related pathways. The possibility that maternal probiotic supplementary during pregnancy might benefit in preventing and treating these diseases need further research. Additionally, the observation of other decreased metabolic functions in the gut microbiota with the use of probiotics, such as peptidases, lipid biosynthesis proteins, and flavone and flavonol biosynthesis was worth noting. Peptidases are responsible for breaking down proteins (69). Its decrease may influence protein breakdown in the gut and nutrient absorption. Likewise, a decrease in lipid biosynthesis proteins suggested a potential alteration in gut microbiota lipid metabolism. This reduction may decrease the production of beneficial compounds by the gut microbiota. Flavonoids are crucial for immune modulation, inflammation reduction, and oxidative stress protection (70). Thus, a decrease in flavone and flavonol biosynthesis might influence gut health and overall immune function, relating to gut microbiota.

The LEfSe analysis revealed that the abundance of *Blautia* and *Subdoligranulum* significantly increased in PG versus CG. *Blautia*, a genus of anaerobic bacteria in the *Lachnospiraceae* family, widespread presence in mammalian feces and intestines (60). *Blautia luti* and *Blautia wexlera*e have garnered significant interest for their probiotic characteristics and notable contributions to alleviating inflammatory and metabolic diseases, alongside their demonstrated antibacterial activity against specific microorganisms (71–74). *Blautia* is negatively correlated with many diseases, including type 1 diabetes, obesity, and Crohn’s disease (75, 76). Studies have shown that *Blautia* is negatively correlated with visceral fat area, which is considered a biomarker of cardiovascular and metabolic disease risk associated with obesity (76). *Blautia* is a major producer of butyrate, a short-chain fatty acid, and experiments have shown that butyrate can have beneficial effects, such as maintaining glucose homeostasis and anti-obesity-related inflammatory characteristics (74, 77, 78). Supplementing triple viable *B. longum*, *L. delbrueckii bulgaricus*, and *S. thermophilus* tablets during pregnancy can increase the presence of *Blautia* in the intestinal tract, which may have beneficial effects on pregnant women during this period. *Subdoligranulum*, found in the human intestinal tract, has the ability to produce short-chain fatty acids. Short-chain fatty acids play various essential roles in maintaining human health, serving as a source of nutrition and energy for the intestinal epithelium. They can protect the intestinal mucosal barrier, reduce inflammation, and enhance gastrointestinal motility (79, 80). Research has shown that *Subdoligranulum* can be beneficial in cases of acute necrotizing enterocolitis by influencing the production of butyrate, a type of short-chain fatty acid (79). The significant increase in *Blautia* and *Subdoligranulum* in the intestinal tract of pregnant women who supplemented with probiotics compared to the non-probiotic group suggests that probiotic supplementation during pregnancy may promote the formation of short-chain fatty acids and potentially have a positive impact on maternal health. Through the analysis of the correlation between differential microbial populations and differential metabolic pathways, it was found that there is a significant correlation between *Blautia* and *Ruminococcus_sp__5_1_39BFAA* in the intestine and multiple intestinal functional metabolic pathways. This indicates a close relationship between these intestinal microbial populations and the functional metabolism of the intestinal microbiota. In this study, high-throughput amplicon sequencing was used to predict the functions of genes in the gut microbiota of pregnant women who took probiotics during pregnancy, revealing differences in functional genes in the metabolic pathways of the gut microbiota under probiotic intervention.

Our study had several limitations. First, it was constrained by its small sample size. Second, the failure to control for potential confounding factors such as diet, lifestyle, and environment. Third, further research should be executed to find the mechanism between gut microbiota and functional diversity in pregnant women.

In conclusion, our findings suggest that probiotic supplementation during pregnancy has a significant impact on functional metabolism. This could potentially open up new avenues for preventing various pregnancy-related complications. This provides new insights into the effects of probiotic consumption during pregnancy on the gut microbiota and offers a convenient method for exploring the potential mechanisms underlying the impact of probiotics on the gut microbiota of healthy pregnant women.

## Data Availability

The original sequencing data presented in this study are publicly available in NCBI under accession number PRJNA1025150.

## SUPPLEMENTAL MATERIAL

Table S1 spectrum.00413-24-s0001.xlsx Level 1 PICRUSt Functional Metabolic Relative Abundance in Each Group.

Table S2 spectrum.00413-24-s0002.xlsx Level 1 PICRUSt Functional Metabolic Relative Abundance in Per Sample.

Table S3 spectrum.00413-24-s0003.xlsx Level 2 PICRUSt the Top 30 Functional Metabolic Relative Abundances in Each Group.

Table S4 spectrum.00413-24-s0004.xlsx Level 2 PICRUSt the Top 30 Functional Metabolic Relative Abundances in Per Sample.

Table S5 spectrum.00413-24-s0005.xlsx Level 3 PICRUSt the Top 30 Functional Metabolic Relative Abundances in Each Group.

Table S6 spectrum.00413-24-s0006.xlsx Level 3 PICRUSt the Top 30 Functional Metabolic Relative Abundances in Per Sample.

ASM does not own the copyrights to Supplemental Material that may be linked to, or accessed through, an article. The authors have granted ASM a non-exclusive, world-wide license to publish the Supplemental Material files. Please contact the corresponding author directly for reuse.
